# Sponge-like sonographic pattern of the spleen in immune dysregulation disorders

**DOI:** 10.3389/fimmu.2026.1868964

**Published:** 2026-06-15

**Authors:** Giorgio Costagliola, Emanuela De Marco, Leonardo Amato, Giulia Roberti, Elena Sabattini, Fabrizio Catena, Mattia Moratti, Gabriella Casazza, Francesca Conti, Rita Consolini

**Affiliations:** 1 Section of Pediatric Hematology and Oncology, Azienda Ospedaliero- Universitaria Pisana, Pisa, Italy; 2 Haematopathology unit, Istituto di Ricovero e Cura a Carattere Scientifico (IRCCS) Azienda Ospedaliero-Univeristaria di Bologna, Bologna, Italy; 3 Unit of Clinical Immunology and Vaccinology, Istituto di Ricovero e Cura a Carattere Scientifico (IRCCS) Bambino Gesù Children Hospital, Rome, Italy; 4 Department of Biomedicine and Prevention, Molecular Medicine and Applied Biotechnology, University of Rome Tor Vergata, Rome, Italy; 5 Pediatric Unit, Istituto di Ricovero e Cura a Carattere Scientifico (IRCCS) Azienda Ospedaliero-Universitaria di Bologna, Bologna, Italy; 6 Department of Medical and Surgical Sciences (DIMEC), University of Bologna, Bologna, Italy; 7 Section of Clinical and Laboratory Immunology, Pediatric Unit, Department of Clinical and Experimental Medicine, University of Pisa, Pisa, Italy

**Keywords:** autoimmune lymphoproliferative immunodeficiencies (ALPID), common variable immunodeficiency (CVID), imaging, inborn errors of immunity (IEIs), lymphoproliferation, malignancy, splenomegaly, ultrasound

## Abstract

**Background:**

Although splenic ultrasound (US) assessment is periodically performed in most patients with inborn error of immunity (IEI), splenic microtexture has not been specifically investigated in this setting. We describe a newly described “sponge-like” splenic microtexture pattern in patients with different IEIs and hematologic immune dysregulation, and discuss its potential implications.

**Methods:**

We present an index case (P1) followed by a cohort analysis including 26 patients with IEIs and 20 controls with chronic immune thrombocytopenia, for whom splenic microtexture data and iconography were available.

**Results:**

In P1, an 11-year-old patient with an autoimmune lymphoproliferative immunodeficiency phenotype, high-frequency splenic US revealed for the first time a “sponge-like” pattern, not visible with standard assessment, characterized by multiple hypoechoic round foci that persisted during long-term follow-up. In the IEI cohort, 46.2% of patients had splenomegaly, and a persistent sponge-like pattern was observed in 30.8% in the absence of infections or malignancies. In the control group, only one of 20 patients showed similar US features, transiently, during acute EBV infection. Immunomodulation (sirolimus, mycophenolate mofetil, rituximab) resulted in a significant improvement or normalization in all cases.

**Conclusion:**

We report for the first time an association between splenic US microtexture features and IEIs. A persistent sponge-like pattern appears to be linked to IEIs/immune dysregulation, and the response to immunomodulation suggests a complex histopathological substrate. Although preliminary, our results suggest that, in patients with other signs of immune dysregulation/dysfunction, identification of this pattern could support earlier suspicion of an underlying IEI, after excluding infections and malignancies.

## 1 Introduction

Inborn errors of immunity (IEIs) comprise a wide range of disorders with different molecular backgrounds and clinical manifestations, ranging from isolated increased susceptibility to severe infections to conditions in which immune dysregulation is the prominent disease feature (1). Most of the described IEIs can present with features of immune dysregulation, including autoimmune cytopenia (AIC), polyclonal (benign) lymphoproliferation (LPD), clonal (malignant) LPD, autoinflammatory manifestations, atopic diseases, enteropathy, and other related phenotypes (2–4). Large cohort studies have shown that LPD is reported in up to 40% of patients with IEIs, manifesting as recurrent or chronic lymphadenopathy and/or splenomegaly (2). Splenomegaly is described with higher frequency in the category of immune dysregulation disorders (such as autoimmune lymphoproliferative syndrome [ALPS] and related conditions), common variable immunodeficiency (CVID) disorders, and others (5).

Considering the incidence of LPD in patients with IEIs, as well as the known incidence of lymphoid malignancies, most patients undergo ultrasound (US) assessment of the lymphatic system, including lymph-node stations and the spleen. In clinical practice, splenic US assessment in patients with IEIs is largely limited to the evaluation of spleen size (diameters, volume) and the search for evident focal lesions, such as abscesses, cysts, or lymphoma localizations. Therefore, the examination is commonly performed with a convex low-frequency probe. By contrast, the analysis of splenic microtexture with high-frequency US (linear probe) is performed only in a limited number of cases, thereby limiting data availability in this setting.

In this paper, we describe a sponge-like US splenic microtexture pattern observed on US in patients with IEIs and hematologic immune dysregulation, through the presentation of a paradigmatic case study, a subsequent cohort analysis, and a potential histopathological correlate of this US finding. Finally, we discuss the most relevant aspects of differential diagnosis in patients presenting with the sponge-like pattern, with the aim of exploring the potential clinical implications of the broader adoption of splenic microtexture assessment using high-frequency US in clinical practice.

## 2 Case presentation

**Figure 1:**
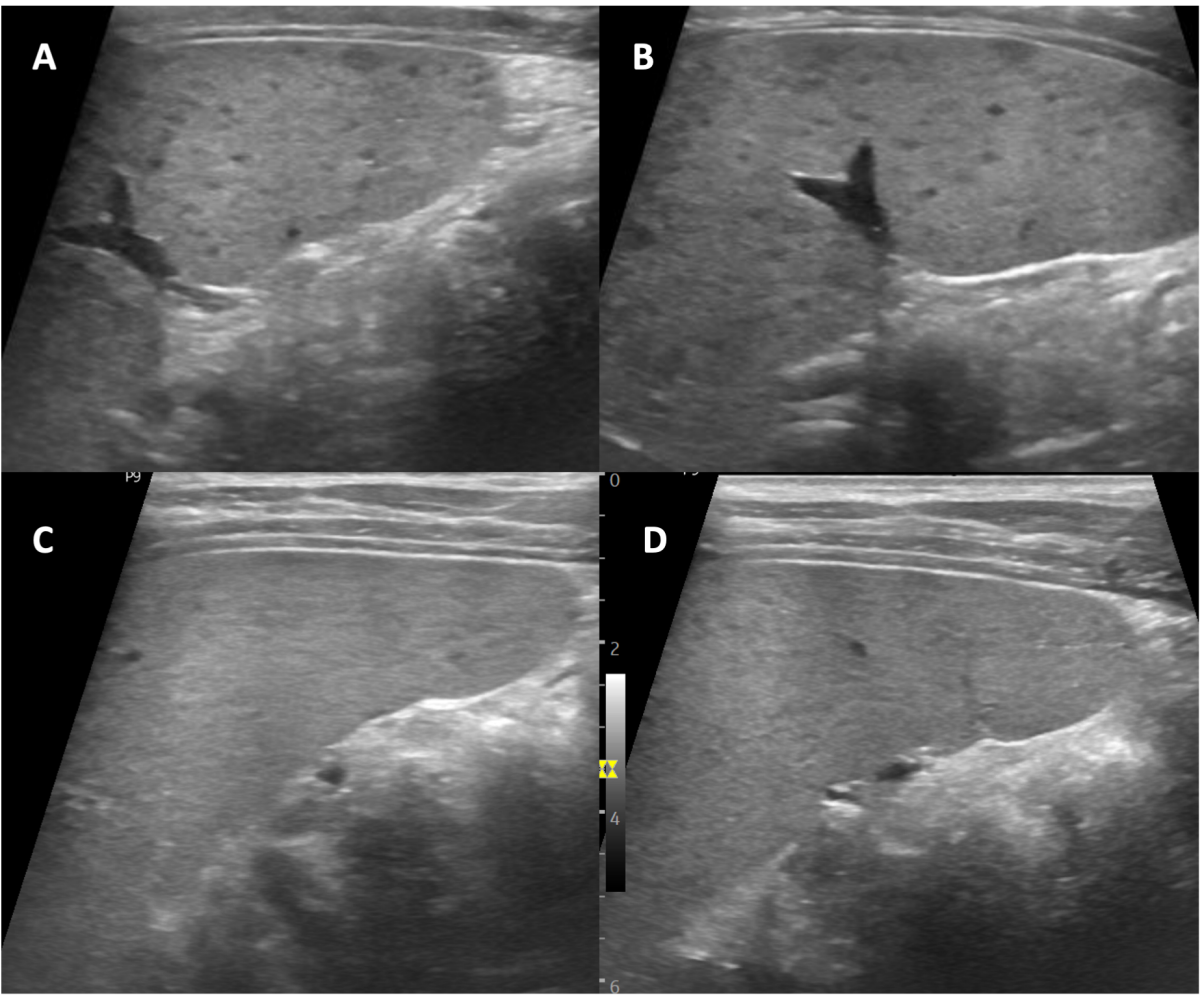
US findings and after treatment with sirolimus in P1. Splenic microstructure of P1 at disease onset **(A, B)**, after 6 months of treatment with sirolimus **(C)**, and after 12 months of therapy **(D)**.

P1 is a boy born from an uneventful pregnancy who came to our attention at the age of 11 with multiple petechiae and left axillary lymphadenopathy. Laboratory investigations revealed thrombocytopenia (subsequently diagnosed as immune thrombocytopenia [ITP]), a positive direct antiglobulin test (DAT) in the absence of overt hemolysis, isolated low IgM levels (≤ 2 standard deviations for age), normal serum B12 levels, and a slight increase in αβ double-negative T cells (αβDNTS: 3.8%). Infectious screening was negative, and bone marrow analysis excluded acute leukemia or hemophagocytic lymphohistiocytosis (HLH). US assessment of the axillary lymphadenopathy revealed a lymph node with increased diameter (4.4 cm) and round morphology, highly suggestive of malignancy, whereas abdominal US with a convex probe did not show pathological findings, and the splenic diameter was within the normal range. However, US assessment with a high-frequency linear probe demonstrated a peculiar pattern characterized by multiple hypoechoic micronodular foci (maximum diameter: 3 mm). These were homogeneously distributed throughout the splenic parenchyma, and no flow was detected with color Doppler. Based on imaging similarities with findings described in a recent study on human deficiency virus (HIV) patients, this was defined as a “sponge-like” pattern (6) (**Figures 1a, b**). During the patient’s clinical course, two lymph-node biopsies were performed, both negative for lymphoma, showing massive hyperplasia (first biopsy) and granulomatous disease (second biopsy). ITP was treated with multiple cycles of intravenous immunoglobulin and corticosteroids, resulting only in a temporary response. Notably, the sponge-like splenic pattern persisted during follow-up, as well as axillary lymphadenopathy.

Given the clinical and laboratory features (chronic LPD, chronic ITP, persistent positive DAT, low IgM levels, increased αβDNTs), the patient was diagnosed as an IEI presenting autoimmune lymphoproliferative immunodeficiency (ALPID) features. Targeted whole-exome sequencing was performed and did not reveal any known pathogenic variant associated with IEIs. In this patient, a likely pathogenic variant in the *NFKB2* gene (c.1061C>T; p.Ser354Phe) was identified. Although this variant is extremely rare in the general population and has a high likelihood of pathogenicity, current knowledge does not allow it to be classified as pathogenic. Therefore, the patient is considered to have an IEI with ALPID-like features and an unknown genetic diagnosis.

After 18 months from disease onset, the patient underwent treatment with sirolimus, which led to the resolution of ITP and stabilization of axillary lymphadenopathy. Interestingly, the sponge-like splenic pattern showed a progressive resolution during therapy (**Figures 1c, d**). Given the peculiarities of this sonographic pattern and its presumptive association with immune dysregulation (also suggested by the response to sirolimus), following this observation, we collected iconography on splenic microtexture using a high-frequency probe in our patients with IEI and immune dysregulation, as well as in patients with chronic ITP.

## 3 Methods of the cohort study

### 3.1 Patient selection

We retrospectively analyzed the records and iconography of patients with IEIs who were followed at the Pediatric Hematology and Oncology Department of Azienda Ospedaliero Universitaria Pisana, undergoing abdominal US with splenic microtexture assessment.

This study includes patients with IEIs defined according to the 2024 International Union of Immunological Societies (IUIS) classification (1) for whom data on US assessment and iconography were available. Diagnosis was made according to clinical and laboratory European Society for Immunodeficiencies (ESID) criteria (7) and/or genetic analysis. Patients already receiving immunomodulatory treatments at the first US assessment were excluded.

### 3.2 Ultrasound assessment

Ultrasound was performed with a GE Healthcare LOGIQ P9 sonographer. The assessment was performed by three different clinicians (E.DM., G.C., F.C.), all certified by the Italian Society for Ultrasound in Medicine and Biology (SIUMB). Each patient included in this study was independently assessed by at least two of these three operators, and agreement between operators concerning the assessment of splenic size, texture, and microtexture was reported. All operators were involved in the clinical care of the included patients and were not blinded to their diagnoses.

Patients were assessed in prone positioning. Splenic structure and size were first evaluated with a convex probe (3.5 MHz), analyzing the bipolar diameter and relation with the other anatomical reference structures, as well as perceptible alterations in splenic texture. Following this, analysis of splenic microtexture was performed using a high-frequency linear probe (7–20 MHz), reporting the presence of focal images, their number, size, and echogenicity.

In all patients with abnormal findings on splenic US, the analysis was repeated after at least 1 month. Patients who started immunomodulatory treatments for the underlying IEI after the first US assessment received periodical US re-evaluations. Changes in splenic size, texture, and microtexture after treatment were analyzed and reported.

### 3.3 Clinical and laboratory assessment

We collected demographic data (age, sex, ethnicity), data regarding the diagnosis of IEIs (age at onset, age at diagnosis, clinical and genetic diagnosis), and treatment received. All patients with abnormal findings on splenic US (splenomegaly or altered macro- or microtexture) underwent an extended work-up to rule out hematological diseases (malignancies, hemoglobinopathies) or infections responsible for the splenic abnormalities. Concerning the diagnostic work-up to rule out infections, patients underwent serology for Epstein–Barr virus (EBV), cytomegalovirus (CMV), *Bartonella henselae*, and *Toxoplasma gondii*, as well as testing for EBV, CMV, and herpesvirus-6 DNA, and an interferon-gamma release assay (IGRA) when a mycobacterial infection was suspected. Other investigations, including HIV testing, were performed based on the clinical suspicion. Investigations to exclude malignancies (bone marrow aspirate/biopsy, lymph nodal biopsy, and others) were also performed based on clinical suspicion.

### 3.4 Definition of a control group

As this study enrolled patients in the hemato-oncology setting, mostly presenting with chronic AIC, we used as a control group a cohort of 20 consecutive patients with chronic ITP, which was defined according to the most updated consensus document (8). Controls underwent the same US assessment protocol used in the patient group.

### 3.5 Informed consent and ethics

This study is approved by the Meyer Hospital Pediatric Ethics Committee, and written informed consent was obtained from the individuals and/or minors’ legal guardians for the publication of any potentially identifiable images or data included in this article.

The patient of the index case (P1) also provided separate informed consent for extended case publication.

## 4 Results

### 4.1 Analysis of the IEI cohort

#### 4.1.1 Demographic data

Overall, clinical data on splenic size and microtexture were available for 26 patients with IEIs and variable diagnoses. These included diseases of the ALPID spectrum (8), humoral IEIs (12), syndromic IEIs (4), and others (2), as detailed in **Table 1**. In this cohort, a genetic diagnosis was available for 53.8% of patients.

**Table 1:** Detailed diagnoses of the included patients with IEI.

Category	Specific diagnosis
Humoral IEIs (12)	CVID-genetic unknown (5)
	TACI deficiency-heterozygous (2)
	NFKB1 mutation (2)
	IRF2BP2 mutation (1)
	unPAD (2)
ALPID (8)	ALPID-genetic unknown (3)ALPS-FAS (1)
	STAT3 GOF (1)
	PKCD (1)
	APDS1 (1)
	XLP-1 (1)
Syndromic IEIs (4)	22q11.2 deletion syndrome (4)
Other (2)	Polyendocrine autoimmune syndrome type 2 (1) Multisystem inflammatory disease-genetic unknown (1)

*22q11-2DS*, 22q11.2 deletion syndrome; *ALPID*, autoimmune lymphoproliferative immunodeficiency; *ALPS*, autoimmune lymphoproliferative syndrome; *APDS*, activated phosphoinositide 3-kinase delta syndrome; *CVID*, common variable immunodeficiency; *GOF*, gain of function; *PKCD*, protein kinase C delta deficiency; *unPAD*, unclassified primary antibody disorder; *XLP*, X-linked proliferative syndrome.

The median age at the first US assessment of microtexture was 13 years. All the patients were treatment-naïve at the first US examination.

In this cohort, splenomegaly was reported in 12/26 patients (46.2%), while a sponge-like pattern was described in eight of 26 (30.8%), with an additional two patients showing a nonhomogeneous splenic texture associated with splenomegaly in the absence of a clear sponge-like pattern. Of these two cases, the first patient was affected by X-linked proliferative syndrome 1 (XLP-1) with EBV-associated HLH and showed a markedly nonhomogeneous splenic texture (also visible with the convex probe), while the other patient was affected by a STAT3 gain-of-function mutation and showed multiple splenic calcifications. The other patients with splenomegaly, in the absence of US texture alterations, were diagnosed with activated phosphoinositide 3-kinase delta syndrome (APDS; *n* = 1), 22q11.2 deletion syndrome (22q11.2DS; *n* = 1), CVID (*n* = 3), and polyendocrine autoimmune syndrome type 2 (*n* = 1).

#### 4.1.2 Details of the sponge-like pattern

Patients with a sponge-like pattern had different underlying diseases, including CVID (*n* = 2), TACI deficiency (*n* = 2), ALPID (*n* = 2), 22q11.2DS (*n* = 1), and protein kinase C delta deficiency (PKCD; *n* = 1), as detailed in **Table 2**. The number and maximum size of the hypoechoic foci varied between patients. However, all patients had a pattern featured by the presence of more than 10 hypoechoic foci (usually > 30) with a diameter ranging between 2 and 6 mm, with no flow on color Doppler and without a defined wall. Notably, in one patient with 22q11.2DS, the number of focal lesions was slightly lower (20–30), but the size of the individual lesions exceeded 6 mm.

**Table 2:** Clinical, laboratory, and US features of the included patients with IEIs and a splenic sponge-like US pattern.

Patient	Diagnosis	Age at first US (years)	Clinical presentation	Relevant laboratory findings	Clinically appreciable splenomegaly	Spleen size (diameter)	Size of the > iso-anechoic lesions (mm)	Treatment	US assessment after treatment
P1	ALPID-(likely pathogenic variant in NFKB2)	11	ITP, lymphadenopathy (chronic, recurrent, requiring two biopsies)	Elevated DNTs, positive DAT, low IgM levels	No	11.7 cm	5 mm	Sirolimus	Marked reduction or disappearance of the lesions
P2	22q11.2DS	3	ITP	Positive DAT, reduced Tregs, elevated CD21low	Yes–severe	13.2 cm	8 mm	Sirolimus	Marked reduction or disappearance of the lesions. Reduction in spleen size (persistence of mild splenomegaly)
P3	TACI deficiency	14	ITP, lymphadenopathy	Positive DAT, anti-dsDNA borderline	No	12.8 cm	4 mm	–	–
P4	TACI deficiency	11	ITP, thyroiditis, Sjogren syndrome	Positive DAT, positive LAC, positive anti-SSa, low IgG	No	12 cm	3 mm	Mycophenolate mofetil	Significant reduction of the lesions
P5	PKCD	17	Diffuse lymphadenopathy (chronic, recurrent), recurrent infections	Pan-hypogammaglobulinemia	Yes	13.5 cm	6 mm	Mycophenolate mofetil, rituximab, cyclosporine	Significant reduction of the lesions
P6	ALPID	4	Lymphadenopathy (chronic, recurrent, requiring two biopsies)	Elevated DNTs, impaired vaccine response	No	9.5 cm	2 mm	Sirolimus	Marked reduction or disappearance of the lesions
P7	CVID	8	Recurrent infections	Pan-hypogammaglobulinemia	No	9.6 cm	2 mm	–	–
P8	CVID	26	Evans syndrome	Pan-hypogammaglobulinemia	Yes	16 cm	2 mm	–	–

*22q11-2DS*, 22q11.2 deletion syndrome; *ALPID*, autoimmune lymphopoliferative immunodeficiency; *CVID*, common variable immunodeficiency; *DAT*, direct antiglobulin test; *DNTs*, double-negative T cells; *ITP*, immune thrombocytopenia; *PKCD*, protein kinase C delta deficiency.

**Figure 2:**
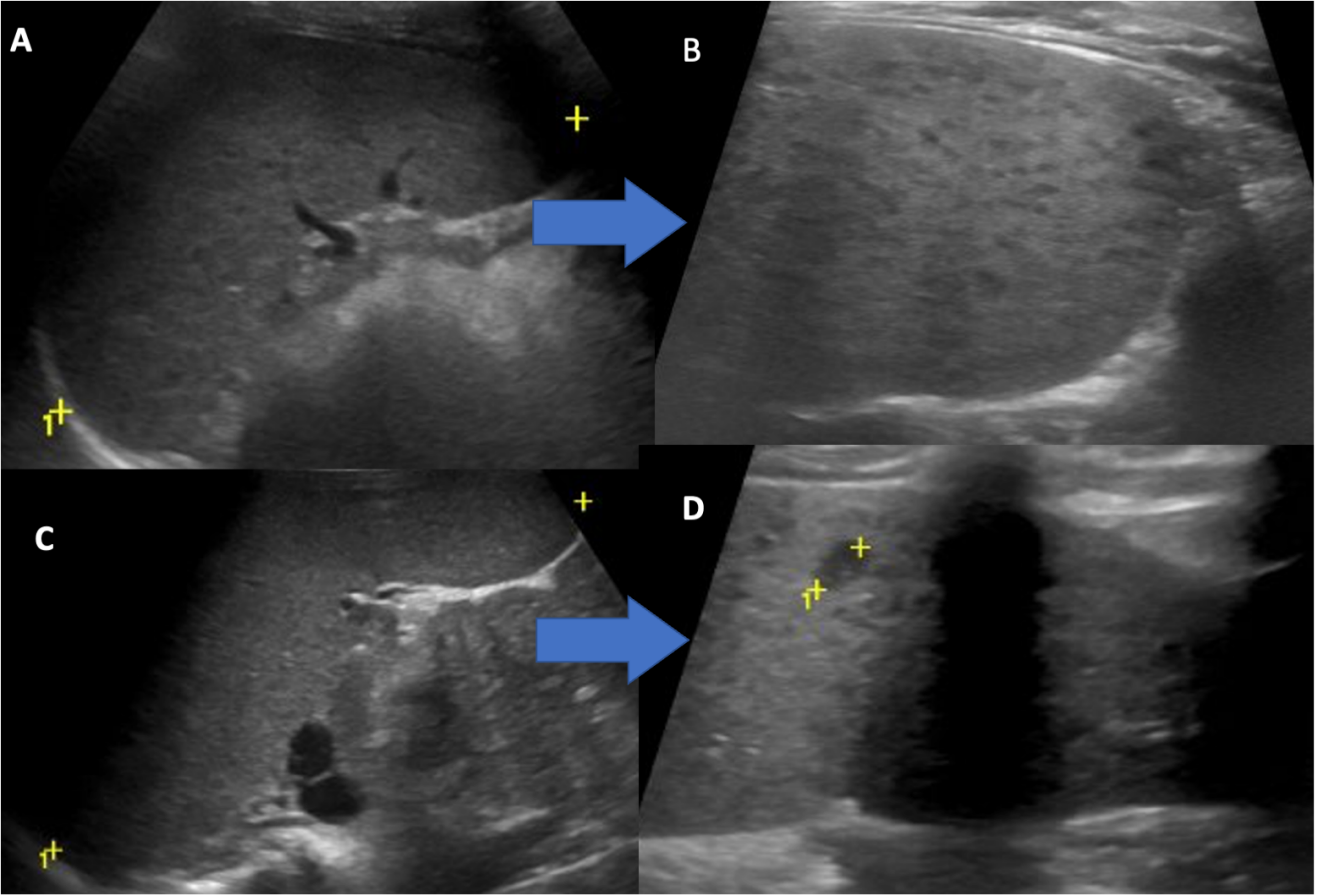
Comparison between findings observed with a convex probe and a high-frequency linear probe in patients with a spleen sponge-like pattern. Two examples of patients are shown in whom US with a convex probe demonstrates normal spleen texture, while high-frequency US reveals a sponge-like pattern [P3: **(A, B)**; P5: **(C, D)**].

In all patients, US with a convex probe did not reveal alterations in splenic texture, and, in five of eight cases, the sponge-like pattern was reported in the absence of evident splenomegaly. **Figure 2** reports an example of paired imaging performed with a convex probe and a high-frequency linear probe.

In all cases, US follow-up confirmed the persistence of the sponge-like pattern after at least 1 month from the first US assessment.

There was a complete reproducibility between the different US operators in the identification of the sponge-like pattern with high-frequency US and in the definition of the spleen diameters and texture with the convex probe (complete agreement in the definition of splenic texture with the convex probe and identification of the sponge-like pattern with high-frequency US, and variability < 1 cm when measuring the spleen diameter).

#### 4.1.3 Clinical associations

All patients with a sponge-like pattern tested negative for acute or recent infections associated with splenic involvement. Malignancies were also excluded through lymph node biopsy (2) or bone marrow aspirate/biopsy (4), based on the clinical picture.

Notably, other signs of immune dysregulation were evident in most cases (seven of eight) and included lymphadenopathy (50%), ITP (50%), Evans syndrome (12.5%), and other autoimmune diseases (25%). Autoantibodies were identified in 75% of cases, and hypogammaglobulinemia was detected in 37.5%. No specific correlations between the sponge-like pattern and clinical, laboratory, or genetic data were identified.

#### 4.1.4 US follow-up and treatment

**Figure 3:**
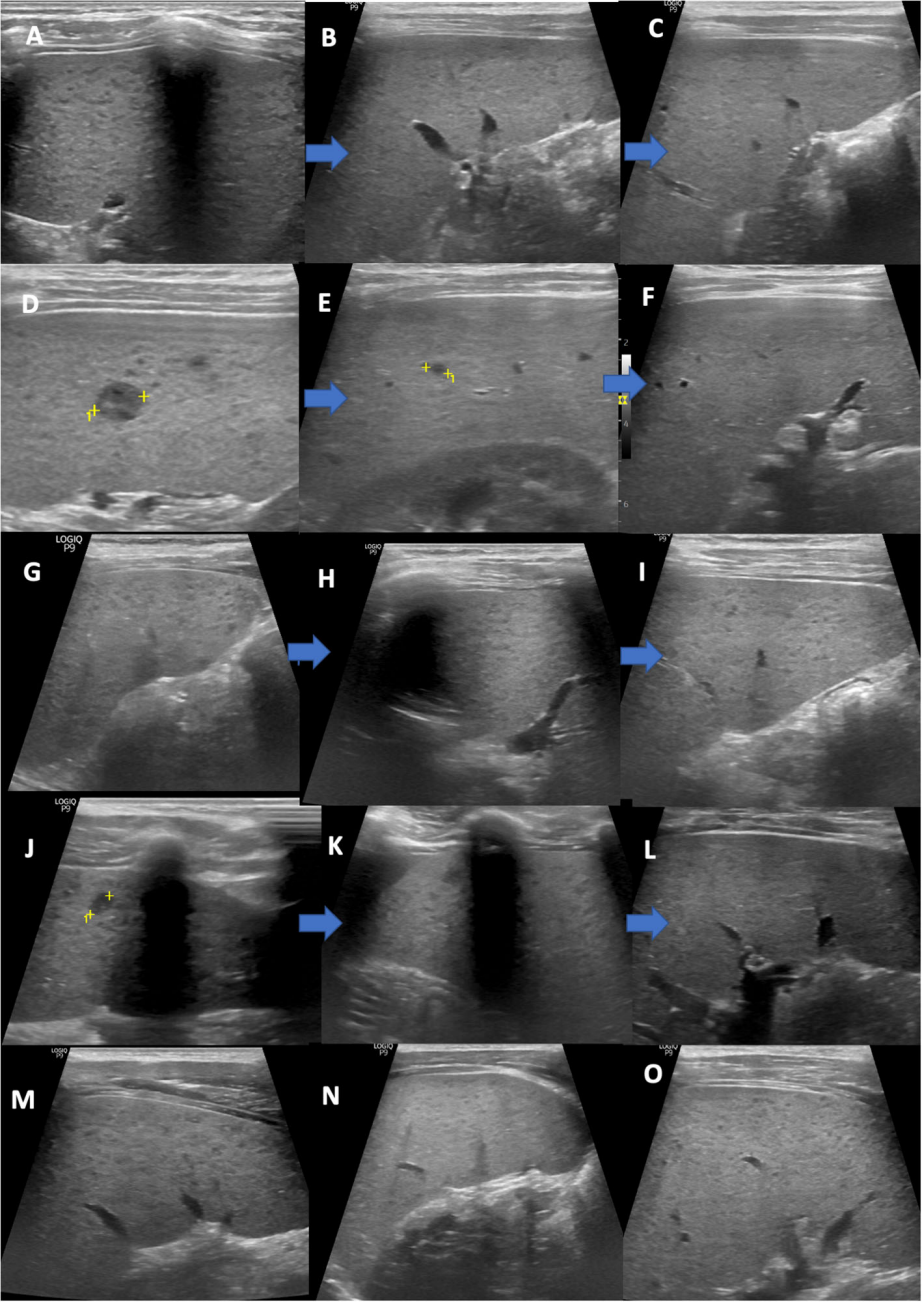
US findings in the other patients with inborn errors of immunity and sponge-like pattern. **(A–C)** Splenic microstructure of a patient with ALPID and unknown genetics at disease onset **(A)**, after 6 months of treatment with sirolimus **(B)**, and after 12 months of treatment **(C)**. **(D–F)** Splenic microstructure of a patient with 22q11.2DS at disease onset **(D)**, after 6 months of treatment with sirolimus **(E)**, and after 12 months of treatment **(F)**. **(G–I)** Splenic microstructure of a patient with a TACI heterozygous mutation at disease onset **(G)**, after 6 months of treatment with mycophenolate **(H)**, and after 12 months of treatment **(I)**. **(J–L)** Splenic microstructure of a patient with PKCD at disease onset **(J)**, after 6 months of treatment with rituximab **(K)**, and after 12 months of treatment **(L)**. **(M)** Splenic microstructure of a patient with a TACI heterozygous mutation. **(N)** Splenic microstructure of a patient with CVID. **(O)** Splenic microstructure of a patient with CVID.

Five patients with the sponge-like pattern received immunosuppressive/immunomodulatory treatments, which included sirolimus (3), rituximab (1), and mycophenolate mofetil (1). All patients treated with sirolimus showed a marked improvement in splenic structure, with no evidence of a sponge-like pattern after at least 6 months of therapy (**Figures 3a–f**). In the other two patients, a significant improvement in splenic microstructure was observed, without full normalization (**Figures 3g–l**).

### 4.2 Analysis of the control group

The cohort of chronic primary ITP included 20 patients. In all cases, a US report of spleen size and texture and iconography was available. All patients in this group received extensive clinical and laboratory work-up to rule out IEIs and other causes of secondary ITP (i.e., connective tissue diseases). The median age of controls at first US was 15.

In this control group, no cases of splenomegaly were reported, consistent with the diagnosis of chronic primary ITP. A spleen texture resembling the sponge-like pattern was transiently detected in only one individual in this group. In this specific case, chronic ITP was associated with autoimmune thyroiditis and mild hypergammaglobulinemia, but the patient lacked clinical and laboratory criteria for the diagnosis of IEIs, and a targeted next-generation sequencing (NGS) panel for IEIs was also negative. Additionally, this patient exhibited an acute EBV infection at the time of US assessment. One month after the first US, following clinical resolution of EBV infection, the splenic microtexture appeared normalized.

In this control group, US follow-up was available in 16/20 individuals and did not show the development of abnormalities in splenic texture.

### 4.3 Histopathological correlation

**Figure 4:**
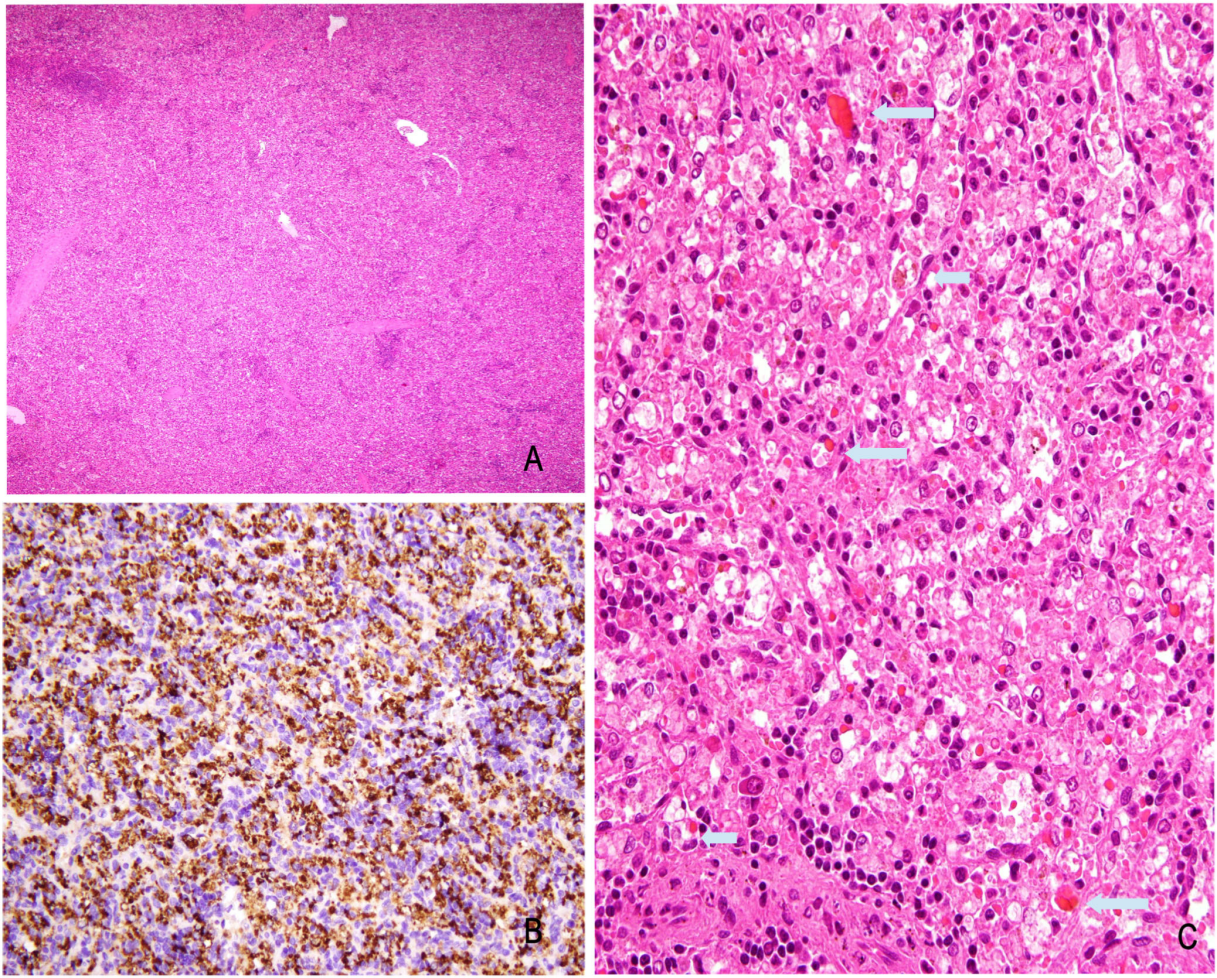
Histological analysis of a spleen of a patient with 22q11.2DS and broad immune dysregulation. **(A)** Hematoxylin–eosin stain showing preserved splenic parenchyma. **(B)** CD68 immunostain highlighting numerous activated macrophages. **(C)** Macrophages showing hemophagocytic activity (arrows), with cytoplasm containing erythrocytes.

An unrelated patient affected by 22q11.2DS and refractory Evans syndrome underwent splenectomy to control the hematologic features. Histological examination of the spleen revealed otherwise unremarkable splenic parenchyma, but with extensive hemophagocytic activity highlighted by strong CD68 positivity. The proliferative index was low, as indicated by a reduced Ki-67 fraction. Only scattered T lymphocytes were identified on CD3 and CD8 immunostaining, while the white pulp contained a minimal residual B-cell population (**Figure 4**). No CD56-positive cells nor T-cell subsets were detected. Finally, *in situ* hybridization for EBV was negative, arguing against an underlying EBV-driven pathology.

## 5 Discussion and future directions

Te field of IEIs with immune dysregulation is in continuous expansion, as new monogenic entities are discovered every year and genotype-to-phenotype correlations are increasingly recognized. One of the most intriguing areas of research in this field is the characterization of the peculiar histopathological patterns observed in the lymphoid tissues of patients with IEIs and LPD. Indeed, it is now increasingly recognized that some patterns, including Castleman−like changes and germinal−center transformation in lymph nodes, are identified with considerable frequency in patients with IEIs (9, 10). However, spleen structure in IEIs is less well characterized, and the correlation between the histopathological data, tissue remodeling, and consequent appearance of lymphoid tissues in imaging investigations has not been thoroughly explored. In this study, we provided an example of how IEI-associated histopathological changes in splenic microtexture can manifest as easily detectable imaging findings. Specifically, this study showed that a “sponge-like” splenic pattern, identified on US with a high-frequency linear probe, can be a feature of different IEIs with hematologic immune dysregulation, even in the absence of overt splenomegaly or other features of LPD.

This US pattern has not previously been reported in association with IEIs in humans. A similar pattern was described in a recently published case series of patients with HIV and was associated with a pathological finding of altered lymphocyte distribution into the white pulp with the formation of lymphocyte infiltrates (6). Interestingly, a sonographic pattern named a “honeycomb spleen”, which presents similarities to the sponge-like aspect in terms of US features, has been described in animals in association with benign LPD (in most cases) and, more rarely, lymphomas (11).

Of note, the histopathological correlation provided in this study is limited to one unrelated case of IEI with broad hematologic immune dysregulation, as splenectomy is performed in IEIs only in extremely selected cases (i.e., life-threatening AIC). Therefore, although the histological analysis in this study revealed extensive macrophage activation, considering the wide pathological background of IEIs, it is reasonable to hypothesize that the histological substrate of this US pattern may not be unique. This could result from a combination of lymphoid hyperplasia (with expansion of the white pulp), granulomatous inflammation with nodular splenic infiltration, disordered architecture (fibrosis, infiltration), and abnormal immune cell accumulation (dysfunctional T and B cells, macrophages). The role of lymphocyte infiltrations in the context of benign LPD is also suggested by the marked improvement in splenic microtexture observed in this cohort after treatment with immunomodulatory agents, including sirolimus, which is commonly used with considerable response rates in IEIs with LPD (12–15). As the number of patients who received immunomodulatory treatment is low, these findings should further be characterized in larger cohorts to better understand variations in US findings among patients treated with different agents.

In addition to these literature data, the interpretation of our findings from a pathological and diagnostic point of view needs to be refined. First, we showed that, as expected considering the heterogeneity in the pathogenesis of IEIs, the sensitivity of this sign is limited, as the sponge-like pattern was observed in less than one-third of the sampled patients. Moreover, this study was performed in a highly selected cohort of patients, predominantly including individuals with other signs of hematologic immune dysregulation, thus potentially overestimating the incidence of splenic involvement. Some conditions (infections, hematological malignancies) can present with splenic US features potentially mimicking the sponge-like pattern (16), while other diseases with splenic involvement (i.e., bacterial and fungal abscesses, hemangiomas, metastatic involvement by solid tumors) have markedly different features in terms of number of lesions, wall characteristics, color-Doppler flow, and echogenicity. To increase the validity of our findings in terms of specificity, we included a control group of patients with non-IEI-associated AIC and also excluded in all included patients other conditions that can be associated with nonhomogeneous splenic textures, such as acute infections (i.e., EBV infection, HIV infection, disseminated mycobacterial infections), hematological diseases (i.e., sickle cell disease), and malignancies (17–21).

As the analysis of the control group emerged only one transient finding of a sponge-like pattern in association with EBV infection, we suggest that the persistence of this splenic pattern, after the exclusion of infections and malignancies, should alert the clinician towards a potential diagnosis of IEI or other conditions characterized by broad immune dysregulation, even in patients without appreciable splenomegaly.

This study has some limitations arising from its retrospective nature, the small cohort size, and potential selection bias, as previously discussed. Additionally, several issues remain poorly explained, such as the association between this US pattern and specific genetic diagnoses or laboratory features, including lymphocyte immunophenotyping. However, although the study is not sufficiently powered to draw definitive conclusions or to support the systematic use of this US pattern as a warning sign for underlying IEIs, it is the first study to directly report an association between the sponge-like spleen US pattern and IEIs. Given the widespread availability of US, its routine application in patients with IEIs, and the technical simplicity of obtaining data on splenic microtexture, this study may open new and interesting perspectives in the setting of IEIs and immune dysregulation disorders, especially concerning the association between pathogenesis, histopathological changes in lymphoid tissues, and imaging findings.

As the sponge-like pattern is not commonly detectable with conventional splenic US assessment using a convex probe, it is reasonable to hypothesize that wider adoption of a targeted high-frequency US spleen assessment in patients with features of immune dysregulation (such as AIC) could increase its identification and ultimately help raise suspicion of an underlying IEI. Considering the promising findings of our analysis, further studies on larger cohorts will hopefully help refine the prevalence of the sponge-like pattern in patients with IEIs and its potential diagnostic role, as well as its prevalence in other non-IEI conditions characterized by broad immune dysregulation (i.e., connective tissue diseases).

## Data Availability

The raw data supporting the conclusions of this article will be made available by the authors, without undue reservation.
